# Please Like Me: Ingratiation as a Moderator of the Impact of the Perception of Organizational Politics on Job Satisfaction

**DOI:** 10.3390/ijerph18147455

**Published:** 2021-07-13

**Authors:** Triana Fitriastuti, Pipiet Larasatie, Alex Vanderstraeten

**Affiliations:** 1 Department of Marketing, Innovation and Organization, Faculty of Economics and Business Administration, Ghent University, 9000 Gent, Belgium; Alex.Vanderstraeten@Ugent.be; 2 Department of Management, Faculty of Economic and Business, Mulawarman University, Samarinda 75123, Indonesia; 3 LPDP Awardee, Gedung Danadyaksa Cikini, Jalan Cikini Raya No. 91A-D, Jakarta 10330, Indonesia; 4 Research Associate, Sustainable Business Management, Oregon State University, Richardson Hall, Corvallis, OR 97331, USA; Pipiet.Larasatie@oregonstate.edu

**Keywords:** organizational politics, perception of organizational politics, political behavior, job satisfaction, ingratiation

## Abstract

Drawing from the negative impacts of the perception of organizational politics (POP) on the literature on organizational outcomes, the model proposed in this study examines a nonlinear relationship of POP on job satisfaction. In a similar way, ingratiation as a moderator variable is tested. Based on a survey of 240 state-owned enterprise employees in Indonesia, this study finds that POP exhibits an inverted U-shaped relationship with job satisfaction. Low and high levels of POP have a negative impact on job satisfaction. Nevertheless, our most intriguing finding is that ingratiation behavior not only strengthens POP’s effects on job satisfaction, but can also alter the direction of the relationship in which its shape is represented by a U-shape. This shape indicates that the employees who engage in high levels of ingratiation as a coping mechanism and adaptive strategy tend to do so when they perceive high degrees of POP. These results are then discussed from a cross-cultural perspective as an attempt to explain the legitimacy of ingratiation in Indonesia.

## 1. Introduction

In an organization, politics may be considered as constituting one of the systems of influence [1] and is often considered as a standard operating procedure [2]. To be effective, organizations need to engage in political behavior and execute these behaviors [1]. For example, politically skilled organization leaders may develop effective social skills [3] alongside the strong networks and social capital necessary to provide resources to their subordinates [4]. However, in order to benefit and protect their self-interests, employees can perform illicit political activities, including coalition-building, backstabbing, favoritism-based pay, and promotion decisions, without considering the welfare of their institution or colleagues [5,6]. Therefore, organizational politics are often perceived as a divisive aspect of the workplace [1].

When employees perceive charged political behavior in their organization environment, there are at least three possible coping mechanism responses that they can use: (1) stay with the organization and attempt to engage with its political behavior; (2) stay with the organization but avoid engaging in the politics; or (3) take an extreme action by withdrawing themselves from the organization [5]. Employees who choose the first way aim to gain a sense of control [7]. To position themselves as pivotal persons, they may use adaptation strategies, such as ingratiation [8]. In popular language terms, reflecting typical ingratiation behaviors include kissing-up, sucking-up, and brown-nosing. These behaviors by employees—best defined as flattery and doing favors—seek to get their boss to like them [9]. As a response to high perceptions of organizational politics, ingratiation behaviors may contribute to satisfaction with supervision and the job of enhancing the relationship between supervisors and their subordinates [10]. As ingratiation is perceived as ubiquitous to some extent in the workplace, this study aims to examine the magnitude of ingratiation, which has a substantive relation to job satisfaction [6,11,12]. We assume that the relationship of perception of organizational politics, job satisfaction, and ingratiation is nonlinear [7,8].

Although there are many studies that have been done in this research domain, most focus on developed countries in North America and Europe [11]. To answer a request by Tsui, Nifadkar, and Ou et al. [12] to test concepts that are well-developed in Western cultures in non-Western settings, as well as a call by Chang et al. [13] to examine the contextual difference on how perceptions of organizational politics influence employee outcomes (i.e., job satisfaction), we chose Indonesia as our study sample. A further reason is that ingratiation is perceived to be impacted by geographic cultures [14,15]

In Indonesia, as in other countries, cultural values are ingrained in society, including the workplace [15]. Indonesians highly respect formal authority derived from hierarchical positions. In line with that, inferiors always need superiors’ instructions and wait for their superiors to make the final decision [16]. At the same time, Indonesians expect that all of them will be consulted when important decisions are made. In Indonesia, ingratiation in the workplace reflects the expression “*asal bapak senang*” or ABS (in English: “as long as the boss is happy”—with the boss described as an older man). This ABS culture is suspected to be a culprit of inefficient bureaucracy in Indonesia [17]. Therefore, we decided to focus on Indonesia’s public sector. Supporting this idea, Vigoda-Gadot and Kapun [18] found that public sector employees view their organizations as more political, unfair, and unjust than private sector employees.

We believe that our study addresses this research gap and provides an opportunity to examine the applicability and validity of concepts on organizational behavior that are primarily developed in Western culture settings. Methodologically, we follow the suggestion to adopt a nonlinear perspective for observing organizational phenomena [19], including investigating nonlinear relationships between political perceptions and job satisfaction [20].

### 1.1. Theoretical Background and Hypothesis Development

#### 1.1.1. Perception of Organizational Politics (POP) and Job Satisfaction

As a common phenomenon in contemporary organizations [21], organizational politics (OP) is defined as activities undertaken within organizations to obtain, develop, and use power and other resources to achieve desired outcomes in an uncertain situation [22]. OP involves intentional acts to benefit, protect, or enhance the individuals’ or groups’ self-interest [23] and influence organizational goals [5,13]. To achieve these goals, employees can demonstrate illegitimate, self-serving political behaviors, including coalition-building, backstabbing, favoritism-based pay, and promotion decisions [5]. As a result, OP can cause disharmony and conflict in workplaces [24], disrupt organizational productivity and performance [21,25], and even be detrimental for individuals, teams, and organizational outcomes [26].

The subjective degree of employee experiences or feelings of politics in their organizational environment is known as perception of organizational politics (POP) [19,27]. POP is found to have both positive and negative outcomes. POP can be considered positive, not just when it appears legitimate, fair, and transparent, but also when it falls within organizational values [28] and when it advances important organizational policies, especially if there is initial disagreement [29]. However, POP can be associated with a variety of dysfunctional psychological health and behavioral outcomes, including organizational commitment [30,31,32], employee engagement, feelings of strain and stress [33], burnout, turnover intentions, as well as decreased levels of job performance and job satisfaction [11,34,35,36].

Many organizational framework studies support the notion of a negative relationship between POP and job satisfaction [13,37]. In this study, we define job satisfaction as ‘‘a pleasurable or positive emotional state resulting from the appraisal of one’s job or job experiences’’ [38] (p. 130). Based on Vroom’s expectancy theory [39], job satisfaction can be low in a politically perceived workplace. Employees can feel insecure and uncertain about reward and punishment mechanisms, as decisions can be politically based [40]. In addition, employees can experience a sense of ambiguity and lack of trust in connecting job effort and performance with reward.

Despite a known negative relationship between POP and job satisfaction, the effects are not always linear [41]. For example, although Harris and Kacmar [42] acknowledged that if increasing POP will increase anxiety levels and intentions to turnover, there is also the possibility that, at some point, it may no longer happen due to the shock of other people’s behavior.

Hochwarter et al. [43] and Rosen and Hochwarter [44] found an inverted U-shaped relationship between POP and job satisfaction. Since employees expect some level of politics to be present in their workplace [45], when politics are moderate, they can better determine the motives and merits of others [20]. Similarly, an inverted-U-shaped relationship is found between perceptions of negative politics and work efforts, depending on the level of rumination about the political behavior [44]. Moderate levels of POP are associated with the highest levels of work effort [44]. In contrast, when levels of politics are very low or very high, it can result in unmanageable stress, uncertainty, and loss of control. These are most often associated with the negative outcomes of POP [15,45,46]. Based on the aforementioned research, we propose a curvilinear, an inverted U-shape, between POP and job satisfaction (negative relationship).

**Hypothesis** **1** **(H1).** *The relationship between POP and job satisfaction will be represented by an inverted U-shape. Specifically, the moderate levels of POP will positively impact job satisfaction while the high and low levels of POP will negatively impact job satisfaction (Appendix A).*

#### 1.1.2. Ingratiation as a Moderator

Although POP and job stress, as a negative job outcome, tended to be positively related, the effect is considered reduced when employees understand the political nature of their organization [46]. This indicates that the effects between POP and job outcomes are varied depending on individual reactions, most likely due to the presence of some moderators [13]. Based on the seminal theoretical work of Ferris et al. [5], numerous studies have attempted to examine the potential moderators between POP and job outcomes, including ingratiation behavior [6,7,11,26].

Ingratiation refers to “a class of strategic behaviors illicitly designed to influence a particular other concerning the attractiveness of one’s personal qualities” [47] (p. 11). Based on this definition, ingratiation can facilitate rewards or decrease the possibilities of individual negative outcomes, supporting the idea of maximizing self-interest [48]. Ingratiation is an effective strategy to shape positive attribution [49] because, in line with human needs, individuals naturally want to be liked [50,51] and appreciated [52]. Therefore, ingratiation can be a natural function for coping with an unfavorable work environment [53]. In the workplace, ingratiatory behaviors are widely used to deal with ostracism [54], abusive supervisors [55], and career barriers [53]. As a result, many terms are induced to conceptualize ingratiation roles, for example, as a neutralizer [55], adaptive strategy [53], adaptive process [56], and strategy enhancer [57].

When POP is perceived to exist and viewed in a negative light [58], employees may engage in an adaptive strategy to mitigate the potential consequences [59]. To provide a clear mechanism, we use the expectancy theory, which has been well-tested in POP studies [24,25,58,59,60,61]. The expectancy theory can be defined as the process of individuals adjusting their behaviors, actions, or tasks as a result of their perception of their environment and expectations [62]. The concept of this theory is that an individual’s motivation adjusts their behaviors as prompted by their perception to gain desired outcomes through expectancy, instrumentality, and valence components [39,63].

According to the expectancy theory, a political workplace environment causes low job satisfaction [58] as employees are unclear about whether their job performance will be positively valued or rewarded [64]. Moreover, employees may believe that their efforts will not be recognized and that their performance rating will remain constant [24]. As a result, employees will likely employ ingratiation strategies that simultaneously serve as adaptation responses to neutralize the unfavorable environment and to obtain a certain benefit [59]. The ingratiator has a certain style that makes others comfortable, resulting in an ability to build relationships and networks easily [65]. Once a network is established, they can gain important information about their workplace and act accordingly. Thus, it enables them to predict, understand, and influence their environments [66]. Similarly, Linden and Mitchell [67] argued that individuals often use ingratiatory strategies when they are highly dependent on other organizational members for completing tasks; to gain information, resources, or other support; and/or when the criteria for the appraisals of job performance and job behaviors are highly subjective.

According to Cook et al. [68], increased political behavior (self-promotion and ingratiation), along with increased organizational political perceptions, will increase both intrinsic (e.g., satisfaction with supervision) and extrinsic job satisfaction (e.g., satisfaction with promotion). Conversely, low political behavior will result in decreased job satisfaction. Moreover, individuals who use ingratiatory behaviors toward supervisors are reported as having a higher level of job and personal satisfaction than those who use these tactics less often [69] (p. 54). Although prior studies find a nonlinear relationship between POP and different job outcomes [6,7,11,26] along with the fact that examining moderators of nonlinear relationships can provide an additional level of precision [70], to our knowledge, there is a lack of research proposing ingratiation as a factor that may foster a nonlinear relationship between POP and job satisfaction.

Most studies examine ingratiation as a moderator [6,68] in linear applications. These research findings reveal that high levels of ingratiation are strongly correlated with diminished negative effects of POP on job satisfaction. The strong correlation of POP and ingratiation from prior research inspired this study to examine a nonlinear relationship, as recommended by Dawson [71] and Cortina [72]. They argued that if the correlation is large (above 0.05), it is essential to conduct a curvilinear test.

Based on the above studies, we assume that the relationship between POP and job satisfaction can be changed to be positive when ingratiation is present. Specifically, when POP and ingratiation are perceived as high, job satisfaction increases from low to high levels. In contrast, when ingratiation is low and moderate, the relationship should remain the same. Tying these insights together, we propose the following hypothesis:

**Hypothesis** **2** **(H2).** *The curvilinear relationship between the negative effect of POP with job satisfaction will be moderated by ingratiation behavior. Specifically, engaging a high ingratiation behavior along with having a high POP level will positively affect job satisfaction.*

## 2. Methods

### 2.1. Study Participants and Procedures

Because our population was Indonesian, we followed the back-translation method (English–Indonesian–English), translated by a professional language institute at Mulawarman University, Indonesia. The survey questionnaire was pre-tested with 40 scholars and practitioners in organizational behavior for language clarity. Based on this test, we reworded some of the potentially ambiguous items.

Since POP varies widely across organizations [11,19], we collected data from 8 multi-sector, state-owned enterprises. We specifically selected organizations that experienced a significant amount of change (i.e., management restructuring and employee downsizing). Under such circumstances, uncertainty and anxiety among employees is perceivably increasing [21,73,74,75], motivating them to establish stronger relationships with their leaders to secure their position and organizational share.

Based on findings that the reality of politics is best understood through the perceptions of individuals [76] and supervisors as one of the most common political players in the workplace [74,77], also following a POP study design in Japan [78], we targeted full-time supervisors with at least two years of experience working within their organization. Most supervisors see organizational politics as a routine part of their work experience [79] while they accomplish organizational and personal objectives [80,81]. As an implicit influence process, political activity serves as a foundational element of most leadership behaviors [2,82]. Political behavior plays a major role in the effectiveness of the decision-making process [83], and the nature and integrity of it has a significant effect on leader emergence and performance [84].

We excluded part-time supervisors because, in Indonesia, as a part of Asian culture, there are significant differences between full-time and part-time workers in social interactions and attitudes at work [85]. We also limited respondents to those with two years’ work tenure or more in their organization, as this is perceived to be the minimum time for a supervisor to understand subtle features of their workplace culture and environment, especially organizational politics [78].

The survey was administered in person by 10 Mulawarman University graduate students. Our population comprises state-owned enterprises located in East Kalimantan, Indonesia. Potential respondents were recruited with assistance from each company’s human resources department head, resulting in contacts with 286 supervisors. Potential study participants were briefly explained the objective of this study and assured that their survey feedback was anonymous and confidential. We also informed them that their participation was voluntary, and they could withdraw from this survey without penalty. Each participant who completed the survey was compensated with a university gift.

From initial contact with 286 supervisors, we obtained responses from 243 supervisors with 240 usable questionnaires after excluding incomplete questionnaires. Just over half the sample (53.75%) were male and the rest were female (46.25%). All participants were aged between 23 and 55 (M = 33 years, SD = 7.64) and had an average job tenure of 12.27 years (M = 6.3, SD = 5.56). Average participants had a relationship with their current manager ranging from 2 to 21 years (M = 4.12 years, SD = 2.67). Participant occupation was composed of 22.5% (n = 54) working in telecommunications, 19.6% (n = 47) in electricity services, 19.6% (n = 47) in banking, 16.3% (n = 39) in construction services, 13.3% (n = 32) in oil and gas, 7.5% (n = 18) in university, and 1.3% (n = 3) in the hotel sector.

### 2.2. Measures

We utilized multi-item scale instruments with modified Likert scales, adapted from previous studies. Responses for POP and job satisfaction variables were assessed using a 5-point Likert-type scale (1 = strongly disagree, 2 = disagree, 3 = neither agree/nor disagree, 4 = agree, and 5 = strongly agree), while responses for ingratiation were measured using a 6-point Likert-scale (1 = strongly disagree, 2 = disagree, 3 = somewhat disagree, 4 = somewhat agree, 5 = agree, and 6 = strongly agree).

To measure Perception of Organizational Politics (POP), we utilized 12 items of instruments developed by Ferris and Kacmar [27]. Example items include “favoritism rather than merit determines who gets ahead around here” and “rewards come only to those who work hard in this organization” as a reverse question. In this study, the Cronbach alpha 0.85 is higher than in prior studies (e.g., 0.81 Li, [86]).

Job satisfaction was measured using 20 questions, as modified by Koh and Boo [87]. The measurement uses five dimensions of job satisfaction: (1) satisfaction with pay; (2) satisfaction with promotion; (3) satisfaction with coworkers; (4) satisfaction with supervision; and (5) satisfaction with work. A higher level of job satisfaction is indicated by a higher mean score. Sample items include “my organization pays better than competitors”, “I enjoy working with the people in my organization”, and “promotions are infrequent in my organizations” (as a reverse question). In this study, the Cronbach alpha for this scale is 0.80.

Ingratiation was measured using the Dimensionality of the Measure of Ingratiatory Behaviors in Organizational Settings (MIBOS) scale developed by Kacmar and Beyerlein [88]. The 15 items were reflected from other enhancement behaviors, opinion conformity, rendering favors, and self-presentation. In this study, the Cronbach alpha for this scale is 0.85.

Control variables were utilized to determine whether ingratiation can emphasize the influence of POP on job satisfaction. To avoid any potential confounding effects on the dependent variables, this study uses demographic variables such as age, gender, job tenure, and relationship with current managers as control variables [43].

### 2.3. Data Analysis

First, we used factor analysis to test the factor structure that loaded significantly on each construct. Second, we performed hierarchical moderated polynomial regression analyses [45,71,89] to test the linear relationship between POP (X) and job satisfaction (Y) and the curvilinear interaction effect of ingratiation (Z) with the following regression equation:

$$Y=\beta_{0}+\beta_{1}X+\beta_{2}X^{2}+\beta_{3}Z+\beta_{4}XZ+\beta_{5}X^{2}Z+C_{0}.$$

Prior to the analysis, we created the interaction and squared terms for computing the regression analysis. Overall, the regression step consisted of six steps. First was entering control variables to ensure our analysis was free from the potential confounding effect. In steps two and three, we tested the linear term, the perception of organizational political (X), and nonlinear terms POP squared ($X^{2}$). In step four, we tested the linear terms of ingratiation as moderator variables, followed by step five, where we computed the interaction between POP and ingratiation (XZ). In the last step, we entered the nonlinear interaction of POP squared with ingratiation ($X^{2}Z)$.

## 3. Results

### 3.1. Validity Test

Before testing the main model, we performed a series of principal component analyses, all with substantive variables (Appendix B). First, to test the 12 items perception of organizational scale, we used principal component analysis with varimax rotation. These scales ranged from 0.05 to 0.79. The items that were loaded below 0.4 were omitted from further analysis. After this step, the Cronbach’s alpha reliability improved slightly from 0.83 to 0.858.

We used a similar method to test 15 items of ingratiation scale. The range of factor loading was from −0.14 to 0.76. Like the prior scales, we omitted items that loaded below 0.4. As a result, the Cronbach’s alpha reliability was increased from 0.70 to 0.80. Lastly, we tested job satisfaction scale factor analysis. The scales ranged from 0.00 to 0.78. By omitting the lowest scale from the general factors, the Cronbach’s alpha reliability was slightly raised from 0.83 to 0.85.

### 3.2. Descriptive Statistics

Table 1 presents the descriptive statistics, including means, standard deviations, and Pearson correlations for all measures. We acknowledge that while Likert questions may be ordinal, since the Likert scales consist of sums across many items, they will be intervals [89]. All the zero-order bivariate correlations are in the expected direction. As shown, POP is positively correlated with ingratiation (r = 0.17, *p* < 0.05). The positive significant correlation POP with job satisfaction (r = 0.15, *p* < 0.05) invoked the need for further investigation. Furthermore, looking to the control variables, age is negatively and significantly correlated with gender (r = −0.22, *p* < 0.01) but positively and significantly correlated with both job tenure (r = 0.35, *p* < 0.01) and current interaction with supervisors (r = 0.27, *p* < 0.01). Gender is negatively significant with current interaction with supervisors (r = −0.14, *p* < 0.05) and job satisfaction (r = −0.14, *p* < 0.05). Tenure as an employee is positively significant with current interaction with supervisors (r = 0.59, *p* < 0.05). Lastly, current interaction with supervisors is negatively correlated with POP (r = −0.13, *p* < 0.05).

### 3.3. Model

This study utilizes Polynomial Regression Analysis to test the curvilinear relationship of perception of organizational politics on job satisfaction and on the moderating effect of ingratiation. The result is summarized in Table 2. As shown in the third step, the POP squared term is significant for job satisfaction (β = −1.18 **, *p* < 0.001). The negative coefficient shown in this step indicates that POP is nonlinear and that the shape of the curve is best represented by an inverted U shape [43,44]. This result supports Hypothesis 1 that the relationship between POP and job satisfaction is represented by an inverted U-shape. Specifically, moderate levels of POP positively affect job satisfaction, while both high and low levels of POP negatively affect job satisfaction.

The result of the moderation effect of the squared POP term with ingratiation is shown in the final step. The interaction between POP and ingratiation behavior explains incremental variance in job satisfaction, with the coefficient of the interaction is positively significant (β = 0.065, ∆$R^{2}$ = 0.05, *p* < 0.000). As the interaction between POP and ingratiation in step 5 has a negative coefficient and the interaction POP square in step 6 is positive, these findings support Hypothesis 2. Ingratiation behaviors moderate the effects of POP on job satisfaction and the relationship is curvilinear. Specifically, for those with high levels of ingratiation, POP will have little effect on job satisfaction as they increase from low to high levels.

**Figure 1:**
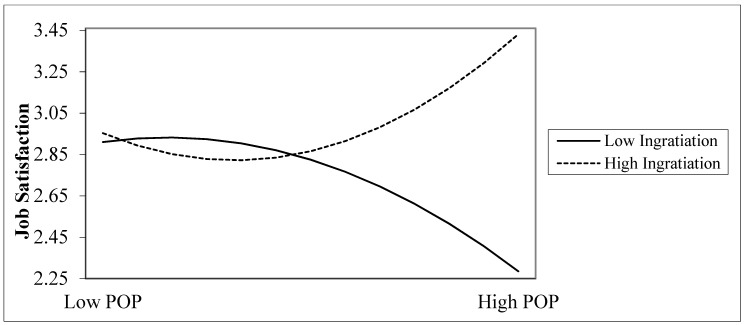
Moderating Effects of Ingratiation on the relationship between Perception of Organizational Politics (POP) and Job Satisfaction.

The significant interactions for high, moderate, and low (−1 SD and +1 SD) values of ingratiation as the moderator are illustrated in Figure 1. The positive coefficient in this step indicates that the relationship is curvilinear, and the curve is represented by a U shape. Figure 1 shows a negative effect of the relationship between POP and job satisfaction among workers with ingratiation as a moderator. When the level of ingratiation is low (1 SD below the mean), the relationship between POP and job satisfaction is negatively significant (t = −1.99, *p* < 0.05). When the level of ingratiation is moderate, the relationship between perceived organization politics and relatedness with job satisfaction is insignificant. Further, high levels of ingratiation (1 SD above the mean) alter the relationship between POP and job satisfaction to be positively significant (t = 4.74, *p* < 0.001).

## 4. Discussion and Conclusions

Our results, showing a significant nonlinear relationship of POP and job satisfaction represented by an inverted U shape, confirm prior studies by Hochwarter et al. [43] and Rosen and Hochwarter [44]. When POP levels are low or high, it can result in unmanageable stress, uncertainty, and loss of control, causing job satisfaction to fall.

Buchanan [77] argues that some employees simply prefer a workplace characterized by a modest level of competitive environment gamesmanship. The most successful employees do not just enjoy playing politics, but they are also reasonably good at it [74]. With moderate levels of POP, employees may have an accurate understanding of internal power structures [90] and, thus, are able to determine the motives and merits of others in their workplace, which facilitates a more manageable work situation and enhances their job satisfaction [43].

Our most intriguing finding is that ingratiation behavior does not just strengthen the effects of POP on job satisfaction, but can also alter the direction of this relationship. Motivated by expectancy theory [39,63], high levels of ingratiation are most essential for the relationship between POP and job satisfaction. This finding reinforces the literature on POP responses [5] and ingratiation studies on POP [6,7,11,26].

In light of the research context, this result demonstrates a cultural workplace phenomenon in Indonesia. The high levels of ingratiation in the relationship between POP and job satisfaction reflects “*asal bapak senang*” or ABS, an expression that means, in English, “as long as the boss is happy”, where the boss is normally described as an older man, as common in the Indonesian public sector. The term highlights that ingratiation behavior seeks to gain benefits for the individual, including shortcuts for career promotion. The results of this study show that the job satisfaction of respondents working in a POP environment is altered after engaging in ingratiation behavior. The more intensely they engage in ingratiation behavior, the more likely that they will be liked by their supervisor. In turn, they gain desirable outcomes, such as fast-tracked promotion. Evidence from a study in Yogyakarta, Indonesia [91] found that local civil servants are aware of the need to engage in political efforts like ingratiation in order to get the first opportunity to be promoted. The political efforts can be carried out openly, but can also be hidden.

We discuss four factors that may explain the legitimacy of ingratiation in Indonesia. First, as a developing country, resources in Indonesia are generally perceived to be limited, which may result in inequality, deprivation, and an uncertain socio-political situation. Based on a study by Pandey [92], in these circumstances, manipulative behaviors like ingratiation are found to be more pervasive. Second, cultural values are ingrained in those workplace settings where Indonesians highly respect formal authority derived from hierarchical positions [15]. The hierarchical structures maintained in traditional and feudal societies may facilitate ingratiation behavior [92].

Another factor that may explain cross-cultural differences in our study is power distance. Based on the concept from Hofstede and Hofstede [93], power distance characterizes the extent to which individuals with less power accept an unequal distribution of power in their organization. Moreover, individuals in societies and organizations with a great power distance will consider impressing their superiors as a legitimate behavior [94]. This is a specific reason why Indonesian employees may engage in ingratiation more intensely; it reflects working in a country with a high power distance [15].

Lastly, the Indonesian score on the assertive dimension is considered at a mid-point, reflecting to the degree to which individuals in a society are assertive, dominant, and aggressive in social relationships [15]. This finding suggests that Indonesians are not encouraged to be assertive. In fact, Hartog [95] states that cultures with low assertive scores highly value not just working cooperatively, but also working harmoniously, and emphasize seniority. An accurate explanation of this finding is that Indonesians are still influenced by their concern for notions like harmony (*rukun*) and father-ism or father-like leadership (*bapak-ism*), factors that prevent them from behaving aggressively and that reinforce behaviors that maintain harmony [96]. These are rooted in cultural values and still exist in the present work environment [97]. Another reason is that cultural values might be related to individualism vs. collectivism [93].

### 4.1. Implications for Management Practice

Theoretically, our results emphasize prior research on the magnitude of POP on job satisfaction [43,44]. To reduce negative effects of POP, organizations must apply fair and transparent management that fosters the job satisfaction of all its employees. In their organizations, managers must put more effort into streamlining communication channels, effectively associating rewards with performance, and carrying out fair punishment.

As prior studies have only focused on linear relationships [43,68], this article also enriches the ingratiation literature. Here, ingratiation is found not only to strengthen the effects of POP on job satisfaction, but can also alter the direction of this relationship. Management should be cautious about employees who climb the career ladder based on ingratiation rather than their real capabilities [97]. Because it is the manager’s duty to appraise work performance and make decisions about promotion, it is important that they are able to see disguised attempts to gain their favor. In other words, managers need to have enough political skill to detect subordinates’ ingratiation behavior. To promote such abilities and skills, training programs can be designed to raise awareness on social cues about understanding situations and multiple behavioral reaction alternatives [98].

As our findings support existing theories on ingratiation as a moderating effect, which explains a parabolic effect, it shows that respondents in our study seek to cope with high POP. Using ingratiation to suppress job dissatisfaction may be one of the best solutions, even though it can achieve different levels of work outcomes. Here, ingratiation behavior can be a double-edged sword, depending on the characteristic of the individuals involved. For example, employees with low POP applying low ingratiation can be ideal since it can result in more stable mental health, although it comes at the cost of career prospects. On the other hand, employees with high POP applying high ingratiation can also be optimal because it can help them climb the ladder, resulting in increased job satisfaction.

### 4.2. Study Limitations and Future Research

We acknowledge several limitations to this study. First, given the relatively small sample size and the fact that the survey was conducted in one region in Indonesia, we cannot readily generalize our findings. Culturally, Indonesia is very complex, comprising 633 ethnic groups [99], each with their own languages and religions. Therefore, to improve the universality of study results, we should conduct similar studies with a broader sample [100].

Second, we acknowledge that there may be differences across the involved sectors. However, our population is state-owned enterprises (SOEs) in which, despite their different sectors, they share the same organizational objectives as an instrument for government income and political control [101]. Therefore, all Indonesian SOEs are managed under one ministry called the Indonesia State Ministry for State-Owned Enterprises (https://bumn.go.id/?lang=en, accessed on 6 July 2021).

Third, we are aware of the common method variance issues associated with self-reported measures in a cross-sectional study. To mitigate the potential problems, we ensured anonymity for each respondent throughout our research design [102]. Additionally, we sought to reduce potential common method bias. All variables were interspersed in the questionnaire so that respondents were unable to recognize any direct relationship between POP, ingratiation, and job satisfaction.

Lastly, our organizational outcomes were limited to only one criterion, that is, job satisfaction. This choice was based on the consideration that, under POP circumstances, job satisfaction is the most quickly affected emotional reaction [21]. Future research should broaden the scope of POP outcomes to include innovation, quality of service, and/or collegiality. POP may also trigger criminal acts, like corruption, because it focuses on achieving personal and group benefits [103].

## Figures and Tables

**Table 1:** Descriptive statistics and zero-order correlations of study variables.

	Mean	SD	1	2	3	4	5	6	7
(1) Age	33.17	7.64	-						
(2) Gender	1.45	0.49	−0.22 **	-					
(3) Job Tenure	6.3	5.56	0.35 **	−0.11	-				
(4) current interaction	4.12	2.67	0.27 **	−0.14 *	0.59 **	-			
(5) POP	2.69	0.66	−0.03	0.01	−0.04	−0.13 *	-		
(6) Ingratiation	4.16	0.43	−0.06	−0.05	−0.04	−0.12 *	0.17 **	-	
(7) Job Satisfaction	3.03	0.62	−0.02	−0.14 *	0.02	0.02	0.15 *	0.12	-

** *p* < 0.01 and * *p* < 0.05.

**Table 2:** Result of Polynomial Regression Analysis for Job Satisfaction of POP on Ingratiation.

Variable	Job Satisfaction
*Step 1*	
Age	−0.002
Gender	−0.121
Job Tenure	−0.006
Tenure with spv	0.013
$∆R^{2}/R^{2}$	0.02/0.02
F	1.42
*Step 2*	
Perception of organizational politics (POP)	0.09
$∆R^{2}/R^{2}$	0.01/0.03
F	1.61
*Step 3*	
Perception of organizational politics (POP)^2^	−1.18 **
$∆R^{2}/R^{2}$	0.06 ***/0.09
F	3.85 **
*Step 4*	
Ingratiation	0.15 ^†^
$∆R^{2}/R^{2}$	0.01 ^†^/0.10
F	3.72 **
*Step 5*	
Perception of organizational politics (POP) * Ingratiation	0.59 ***
$∆R^{2}/R^{2}$.	0.05 ***/0.15
F	5.20 ***
*S* *t* *ep 6*	
Perception of organizational politics (POP)^2^ * Ingratiation	0.65 ***
$∆R^{2}/R^{2}$	0.05 ***/0.20
F	6.75 ***

*** *p* < 0.001, ** *p* < 0.01, * *p* < 0.05, ^†^ *p* < 0.10.

## Appendix B

**Items**	**POP**	**Ingratiation**	**Job Satisfaction**
Employees are encouraged to speak out frankly even when they are critical of well-establish ideas. *(RS)*	0.62		
Whereas a lot of what my supervisor does around here (e.g., communicates and gives feedback, etc.) appears to be directed et helping employees, it is actually intended to protect himself/herself	0.69		
There is no place for yes-men around here; good ideas are desired even when it is means disagreeing with superiors. *(RS)*	0.71		
When my supervisor communicates with me, it is to make himself/herself look better, not to help me	0.77		
The performance appraisals/ratings people receive from their supervisors reflect more supervisor’s ‘own agenda’ (e.g., likes and dislikes, giving high or low ratings to make themselves look good, etc.) then the actual performance of the employee.	0.71		
Managers in the organization often use the selection system to hire only people that can help them in their future or who see things the way they do	0.80		
If a co-worker offers to lend some assistance, it is because they expect to get something out of it (e.g., makes them look good, you owe them a favor now, etc.), not because they really care.	0.56		
My co-workers help themselves, not others.	0.59		
Connections with other departments are very helpful when it comes time to call in a favor	0.53		
I have seen people deliberately distort information requested by others for purposes of personal gain, either by withholding it or by selectively reporting it.	0.55		
Tell him or her that you can learn a lot from his or her experience		0.46	
Exaggerate his or her admirable qualifies to convey the impression that you think highly of him or her		0.51	
Ask your supervisor for advice in areas in which he or she thinks he or she is smart to let him or her feel that you admire his or her talent		0.54	
Look out for opportunities to admire your supervisor		0.77	
Go out of your way to run an errand for your supervisor		0.68	
Offer to help your supervisor by using your personal contacts		0.73	
Volunteer to be of help to your supervisor in matters like locating a good apartment, finding a good insurance agent, and the like		0.56	
Spend time listening to your supervisor’s personal problems even if you have no interest in them		0.63	
Volunteer to help your supervisor in his or her work even if it means extra work for you		0.73	
My organization pays better than competitors			0.42
If I do a good job, I am likely to get promoted			0.45
The people that I work with do not give me enough support *(RS)*			0.52
In my organization, when I ask people to do things, the job dets done			0.67
I enjoy working with the people in my organization			0.82
In my organization, I work with responsible people			0.79
The managers I work for back me up			0.51
The managers I work for are competent			0.43
My job is interesting			0.72
I feel good about the amount of responsibility in my job			0.73
I would rather be doing another job *(RS)*			0.67
I get little sense of accomplishment from doing my job *(RS)*			0.51
Items and factor loadings; R = reverse item.			
